# Family Caregiving Disrupted by COVID-19: Overcoming Challenges Through Resilience and Flexibility

**DOI:** 10.1093/geroni/igab046.1735

**Published:** 2021-12-17

**Authors:** Mary Wyman, Laura Wray

## Abstract

The impacts of the COVID-19 pandemic have been global and pervasive – yet only partially understood. Older adults and their family caregivers have experienced profound and unprecedented challenges as a result of COVID-19. This symposium features research on these disruptions and the creative adaptations undertaken in response. Four presentations present a variety of geographic regions, caregiving settings, and research focal points. Avidor and Ayalon present findings from interviews with family caregivers of residents in long-term care, highlighting the issues faced as a result of dramatic shifts in policy and procedures in support of pandemic infection control. Gum and colleagues focused on how service access has changed for community-dwelling older adults and their families during the pandemic, and how agencies may best leverage the flexibility of caregivers to adapt service provision. Boucher et al. compare the challenges faced by family caregivers during COVID-19 with those faced during a natural disaster, and highlight unique differences in service access for caregivers of military veterans. Finally, Ko and co-authors share their experience with the implementation of a technology intervention to reduce caregiver stress as part of a research trial, and how the protocol adjustments necessitated by COVID-19 revealed the unique potential of such technologies to support caregivers under isolated conditions. The presenters will focus on themes of resilience and lessons learned for health care systems, service agencies, and society to best support family caregivers in challenging circumstances moving forward.

